# Commentary: Basic Research in HIV Vaccinology Is Hampered by Reductionist Thinking

**DOI:** 10.3389/fimmu.2016.00042

**Published:** 2016-02-10

**Authors:** Mike R. King

**Affiliations:** ^1^Division of Health Sciences, Bioethics Centre, University of Otago, Dunedin, New Zealand

**Keywords:** HIV vaccines, rational vaccine design, reductionism, reverse vaccinology, ethics, distributive justice, philosophy of science

Marc Van Regenmortel argues that the cumulative weight of evidence supports the conclusion that structure-based reverse vaccinology of an anti-HIV vaccine is highly unlikely to succeed (1). He argues that the reductionism inherent in this approach ignores important immunological properties that emerge at higher levels of organization in biological systems, and is myopic in its view of immunogenesis, focusing unduly on one aspect of this complex integrated process (the structure of viral epitopes bound to neutralizing monoclonal antibodies). Van Regenmortel concludes that failure of vaccine development to account methodologically for this complexity explains the failure to date of structure-based reverse vaccinology to develop a vaccine capable of raising broadly neutralizing antibodies against the HIV virus.

The situation Van Regenmortel describes is striking. If his argument is correct, a costly research program is being pursued, which is likely to fail in its beneficial, ultimate aim – the development of a successful vaccine. If properly deployed, an effective vaccine could dramatically reduce the infection rate, estimated at around two million new infections in 2014, and the harm caused by the global HIV epidemic which in 2014 affects some 36.9 million people worldwide (2). Given the scope of this harm, the potential benefit of HIV vaccine development is great, much of which would accrue to disadvantaged groups, such as the population of Sub-Saharan Africa (2). If researchers are devoting scarce resources to ineffective research programs when those same resources could be used more effectively to pursue this good via other means, a moral wrong is occurring. In broad terms, the wrong is a failure of distributive justice, which allows a risk of significant harm to others to persist.

Van Regenmortel’s argument that reverse vaccinology is inappropriate for HIV vaccine development rests on the claim that reverse vaccinology relies on incorrect theoretical assumptions about the immune response. He claims that this is the most reasonable conclusion to draw from, *inter alia*, the occurrence of a multitude of negative results from attempts to derive successful vaccine immunogens from candidate HIV-1 epitopes, which bind broadly neutralizing antibodies against HIV. I will raise two challenges for this argument.

First, the complexity of scientific theories and experimentation is such that it is very difficult to conclusively attribute negative results (such as those Van Regenmortel presents) to the falsity of particular theoretical assumptions reflected in methodology. Also, it is unclear what should be taken from Van Regenmortel’s claim that the failure of “hundreds of attempts” to develop an effective HIV vaccine using reverse vaccinology shows the falsity of the reductionism that underlies the experiments, and militate in favor of an alternative approach (1). If many different research groups each make such attempts simultaneously and/or there is a lack of adequate coordination and information exchange between them – as is arguably the case in HIV vaccine research – many failures may arise from slow development of experimental knowledge (3). Alternatively, if few groups have the opportunity to learn from and not repeat each other’s mistakes, consistent lack of success points more strongly toward false assumptions underlying the research. In short, it is reasonable to question the correct inferences to be drawn from a critical assessment of the evidence.

Whether they are used to support or undermine a theory, the strength of convergence arguments depends on the degree to which evidence converges on support for, or undermining of, the theory in question, and the amount of this evidence, critically considered (4). The first objection I mentioned questions the degree to which any experimental evidence converges on – and only on – one theory or philosophical assumption (and example of the problem of underdetermination in scientific theory) (4, 5). The second questions the meaningfulness of the amount of this evidence. These objections are not decisive, and there is much more that could be said, both for and against the convergence argument that concludes Van Regenmortel’s review. I present them to show that commitment to methodological approaches in science, and the theories and assumptions that underlie them, can often be defended even in the face of what must be acknowledged as significant evidence of their lack of success, both in this case, and more generally. This is sometimes a matter of most significance for scientists, and not others. However, in HIV vaccinology, the well-being of millions of people, the majority of which come from populations with great material need depends on the efficient use of correct theory and method.

What I hope is clear from this discussion, and particularly Van Regenmortel’s article, is that immunology, and the biosciences more broadly, are permeated with philosophical assumptions and that practical, methodological questions can hinge on them. What is striking about the area of vaccinology is what is at stake morally, as opposed to purely epistemically, when research is unproductive. Van Regenmortel provides a strong argument that this lack of productivity in the case of HIV reverse vaccinology is, at least in large part, due to naïve commitment to incorrect reductionist assumptions. I find his argument persuasive despite the objections I have mentioned. I mention them to show that even those who disagree with his argument must have some philosophical knowledge and skill in order to engage with his reasoning and defend their practice, if they choose. More generally, a reasonable understanding of philosophy, or at least philosophy of science, should be part of the skills of any scientist. If those applying reverse vaccinology to HIV cannot defend their approach against the arguments Van Regenmortel presents, and if they value rationality, they must change their practice appropriately. Irrational, unproductive, scientific practice in this case is not just wasting resources, it is also sacrificing the well-being of those who might otherwise be spared HIV infection by earlier vaccine development. If the reverse vaccinology approach for HIV vaccine development is not defensible philosophically, it is not defensible morally.

## Author Contributions

The author confirms being the sole contributor of this work and approved it for publication.

## Conflict of Interest Statement

The author declares that the research was conducted in the absence of any commercial or financial relationships that could be construed as a potential conflict of interest.
